# The Annalist

**Published:** 1848-04

**Authors:** 


### THE MEDICAL EXAMINER.

### PHILADELPHIA, APRIL, 1848.

### THE ANNALIST.

We regret to observe an editorial article in this excellent periodical, of the 15th ult., animadverting, in language which we are not accustomed to see in its pages, on the bibliographical notice of "Tracts on Generation" contained in our last number. As it is our desire on all occasions to avoid giving just cause of offence, in anything we may promulgate, we have looked carefully over the obnoxious article, but without discovering anything that ought, as far as we can perceive, to be regarded as illiberal or unfair criticism; nor have we been enlightened in this search by any disproof of the facts or reasonings in the "Notice" afforded by our contemporary.

By turning to the article in the "Examiner," page 190, the reader will find that the introductory portion is occupied with a just rebuke of a practice which all must admit is "more honoured in the breach than the observance;" and whether the rebuke properly applies or not to the "letter" commented on, must be left to the reader to determine. Our brother thinks the eulogies lavished upon "M. Agassiz, the distinguished naturalist," have not been "extravagant," or wanting "in true dignity," because, in his opinion, it was performing a "simple act of justice." Nothing has been said in our pages to detract from the merits of M. Agassiz, or to deny the justice of all that is alleged of "his great scientific attainments, his pleasing delivery, his amiable manners, and his retiring modesty;" but the "good taste" of such commendation, uttered in the very ear of the object, whether just or not, was and is denied. Beside the "literary hero" who was so lauded and bepraised on his visit to this country some years ago, we have a pretty vivid recollection of the unmeasured commendations of the press in "New Amsterdam," bestowed on "the pleasing delivery" and "almost perfect system of Mnemonicks of Professor Gouraud." If our brethren of New York have not learned wisdom from such experience, they should at least have some charity for those who are less ready than themselves to yield to impulses, although not less ready to appreciate and acknowledge true merit.

The Editor of the "Annalist" confesses that "it may be that the term of '*a German science*,' as applied to ovology, is somewhat hyperbolical;" and we have no doubt that both his readers and ours will agree with him in this. In some future number we shall expect to find him admitting also that the writings of Bischoff do not need to be endorsed by Agassiz or any one else, and that those who translate his productions, should not require the supervision of the distinguished naturalist to ensure the accuracy of their performance.

But what has the editor of the Annalist to do with all this? He is neither author, translator, nor sponsor: why then does *he* take us to task? Who has constituted *him* the censor of other journalists? "Whether this be courteous or just," and whether it be expedient for one journalist to take cognizance of the labours of another, we leave it to "the sober second thought" of "our esteemed confrère" to determine. It is true the editor thinks himself particularly implicated in this matter for the following reason: "Now, as we have seen no eulogies of M. A. in any of our Southern or Eastern exchanges, in which sections of our country only, we believe, M. A. has lectured; and as *we have* spoken in very high terms of this gentleman, both in his social and scientific character, we venture to assume this reproach for ourselves." A very unnecessary assumption, we think. It was not alleged in the Examiner that the laudations were chiefly or at all confined or even contained in medical journals, and if the editor has "seen no eulogies of M. A. in any of his Southern or Eastern exchanges," it is certainly no proof that such never appeared in other publications: but even in his "exchanges," if he will look a little more closely, we think he will yet find some expressions of that kind. Suppose it to be as he says, that no laudatory notices of M. A. have appeared in any journal but the Annalist, ought not that fact to be regarded as evidence of the correctness of the criticism in the Examiner? Does it not suggest to the editor that he may have been injudicious? Surely he does not wish to be regarded as impliedly charging the conductors of his "Southern and Eastern exchanges" with incapacity to discover or unwillingness to acknowledge great virtues and abilities! And yet such would seem to be the natural inference.

In conclusion, we cannot avoid the expression of our regret that our brother should have given occasion to these remarks by his interference in a matter in which, with all due respect, we must be permitted to think he had no direct concern. Nevertheless, if on examination of the remarks to which he has objected, we had found any such expressions as "*a verbal cavilling based upon a misinterpretation, or a wilful misrepresentation of a single sentence, and a disposition to impute ignorance*," *&c.*, we should indeed have thought the "tone" objectionable, and that our "critical supervision" should "have modified or excluded" such remarks.

